# Multivariate generalized mixed-effects models for screening multiple adverse drug reactions in spontaneous reporting systems

**DOI:** 10.3389/fphar.2024.1312803

**Published:** 2024-01-16

**Authors:** Masahiko Gosho, Ryota Ishii, Tomohiro Ohigashi, Kazushi Maruo

**Affiliations:** ^1^ Department of Biostatistics, Institute of Medicine, University of Tsukuba, Tsukuba, Japan; ^2^ Department of Biostatistics, Tsukuba Clinical Research and Development Organization, University of Tsukuba, Tsukuba, Japan

**Keywords:** database, drug-drug interaction, proportional reporting rate, reporting odds ratio, signal detection

## Abstract

**Introduction:** For assessing drug safety using spontaneous reporting system databases, quantitative measurements, such as proportional reporting rate (PRR) and reporting odds ratio (ROR), are widely employed to assess the relationship between a drug and a suspected adverse drug reaction (ADR). The databases contain numerous ADRs, and the quantitative measurements need to be calculated by performing the analysis multiple times for each ADR. We proposed a novel, simple, and easy-to-implement method to estimate the PRR and ROR of multiple ADRs in a single analysis using a generalized mixed-effects model for signal detection.

**Methods:** The proposed method simultaneously analyzed the association between any drug and numerous ADRs, as well as estimated the PRR and ROR for a specific combination of drugs and suspected ADRs. Furthermore, the proposed method was applied to detect drug-drug interactions associated with the concurrent use of two or more drugs.

**Results and discussion:** In our simulation studies, the false-positive rate and sensitivity of the proposed method were similar to those of the traditional PRR and ROR. The proposed method detected known ADRs when applied to the Food and Drug Administration Adverse Event Reporting System database. As an important advantage, the proposed method allowed the simultaneous evaluation of several ADRs using multiple drugs.

## 1 Introduction

During the clinical development of new drugs, collecting sufficient information on drug safety poses a considerable challenge. Hence, spontaneous reporting systems are crucial sources for post-marketing drug safety surveillance. Importantly, these systems are commonly used to detect suspected adverse drug reactions (ADRs) and generate potential ADRs in real-world settings. Since the 1960s, regulatory authorities such as the US Food and Drug Administration (FDA) have established databases for spontaneous reporting.

When assessing drug safety using spontaneous reporting system databases, quantitative signal detection methods can be valuable for identifying the relationship between a drug and suspected ADR, given the considerable amount of data obtained. This data mining approach is crucial for the early detection of safety signals and for generating hypotheses regarding new ADRs. Several methods, including the proportional reporting rate (PRR) (Evans et al., 2001), reporting odds ratio (ROR) (Rothman et al., 2004), Bayesian confidence propagation neural network (BCPNN) (Bate et al., 1998), and multi-item gamma Poisson shrinker (MGPS) (DuMouchel, 1999), have been proposed and employed by regulatory authorities for signal detection. These methods typically assess disproportionality in the observed and expected numbers of counts for specific combinations of a drug and suspected ADRs. Thus, if the ratio of the observed count to the expected count (henceforth, the O/E ratio) estimated using these methods is far from 1, it is considered a signal. Although the performance of these methods has been extensively evaluated and compared (van Puijenbroek et al., 2002; Kubota et al., 2004; Almenoff et al., 2006; Matsushita et al., 2007; Hochberg et al., 2009; Ahmed et al., 2010; Bunchuailua et al., 2010; Chen et al., 2015), no gold standard method has been established worldwide.

Unlike the BCPNN and MGPS, the PRR and ROR are easy to calculate and interpret. The PRR is a simple risk ratio (or relative risk), while the ROR is a simple odds ratio derived from a 2 × 2 contingency table (Table 1), with both measurements closely related to statistical models occasionally used for signal detection. Considering Poisson regression models, the parameter estimates in the model yield the PRR, which is the reporting ratio of drug use to non-use. Likewise, the ROR can be estimated using a logistic regression model. In particular, these models help assess drug-drug interactions (DDIs) during the concurrent administration of two or more drugs (Thakrar et al., 2007). By including a statistical interaction term in the model, the presence of DDIs can be evaluated using a spontaneous reporting system (van Puijenbroek et al., 1999; van Puijenbroek et al., 2000). Importantly, these modeling approaches can detect only one ADR, and multiple models need to be constructed for each ADR to estimate the PRR and ROR of various ADRs. For example, to evaluate 100 types of ADRs, 100 regression models must be constructed with each ADR as a response variable.

**TABLE 1:** Two-by-two contingency table for summarizing the specific ADR reported in the target drug.

Number of events (incidence probability for j th ADR)	Specific ADR j	All others	Total
Target drug, i	$n_{ij}$ $\left(p_{ij}\right)$	$n_{i\cdot }-n_{ij}$	$n_{i\cdot }$
All others, $i^{-}$	$n_{j}-n_{ij}$ ( $\left.p_{i^{-}j}\right)$	$n-n_{i\cdot }-n_{\cdot j}+n_{ij}$	$n-n_{i\cdot }$
Total	$n_{\cdot j}$ ( $\left.p_{\cdot j}\right)$	$n-n_{\cdot j}$	n

ADR, adverse drug reaction.

As another approach for detecting DDIs, Norén et al. (2008) proposed a criterion using the O/E ratio of the number of reports for the ADR for a combination of two drugs. Gosho et al. (2017) also proposed a criterion based on chi-square test statistics to measure the discrepancy between the observed and expected number of reports. Although these methods have been effectively reviewed and compared (Noguchi et al., 2019; Noguchi et al., 2020), the detection of DDIs between three or more drugs is not possible. Moreover, similar to the analysis using regression models, the methods can detect only one ADR, and multiple analyses are required to assess each ADR.

In the present study, we propose a novel, simple, and easy-to-implement method using Poisson and logistic mixed-effect models for signal detection. The proposed method could simultaneously analyze the relationship between any drug and numerous ADRs and estimate the PRR and ROR for a specific combination of drugs and suspected ADRs. Furthermore, the proposed method could be applied to detect DDIs during the concurrent administration of two or more drugs. We also provide a sample SAS code for implementing the proposed method.

## 2 Methodology

### 2.1 Notation

Spontaneous reporting systems include multiple drugs and ADRs in each report. This information can be summarized in a 2 × 2 contingency table, as shown in Table 1. We used I drugs and J ADRs. Here, $n_{ij}$ is the number of events reported for the i th drug ( $\left.i=1,\ldots ,I\right)$ and j th ADR $\left(j=1,\ldots ,J\right)$ ; $n_{i\cdot }$ is the total number of events reported with the target drug i ; $n_{\cdot j}$ is the total number of events reported with specific ADR j ; n indicates the total number of ADRs reported with any drug; $p_{ij}$ is the incidence probability for the j th ADR with the i th drug. Let $i^{-}$ denote all other drugs except the target drug i .

### 2.2 Standard strategy and signal detection methods

Typically, signal detection is used to assess disproportionality in the observed number of counts, $n_{ij}$ , and the expected number of counts, $E_{ij}$ , for a specific combination of drug i and ADR j . $E_{ij}=n_{i}n_{j}/n$ is defined as the expected number of counts under the null hypothesis, with no association between the i th drug and the j th ADR. O/E ratios were evaluated using several methods. The direct estimator of the O/E ratio is the relative reporting ratio, defined as:

$$\frac{n_{ij}/n_{i}}{n_{j}/n}=\frac{n_{ij}}{E_{ij}}$$

The PRR was calculated as the ratio of the proportion of the ADR j reported with drug i to the proportion of the same ADR reported with all other drugs combined:

$$\frac{n_{ij}/n_{i}}{\left(n_{j}-n_{ij}\right)/\left(n-n_{i}\right)}$$ (1)

The PRR can be interpreted as a measure of the reporting rate, with and without target drug i . In addition, PRR is considered an approximation of the relative reporting ratio, given that $n_{ij}\ll n_{j}$ and $n_{i}\ll n$ in almost cases. If PRR = 1, the absence of an association between the i th drug and the j th ADR can be assumed.

The ROR was calculated as the ratio of the odds for ADR j reported with drug i to the odds that the same ADR was reported with all other drugs combined, as follows:

$$\frac{n_{ij}/\left(n_{i}-n_{ij}\right)}{\left(n_{j}-n_{ij}\right)/\left(n-n_{i}-n_{j}+n_{ij}\right)}$$ (2)

If the lower limit of the 95% confidence interval (CI) for PRR is greater than 1, the relationship between the target drug and specific ADR was detected as a signal; the same was applied to ROR in Eq. 2.

### 2.3 Poisson mixed-effect model and PRR

We assumed that the random variable $n_{ij}$ follows a Poisson distribution, expressed as $n_{ij}\;∼\text{ Poisson}\left(n_{i}p_{ij}\right)$ , where $p_{ij}$ is the incidence probability of ADR j when drug i is used (Table 1). The probability function is expressed as

$$\mathrm{Pr}\left(n_{ij}\left|\;\right.\lambda_{ij}\right)=\frac{\exp\left(-\lambda_{ij}\right){\lambda_{ij}}^{n_{ij}}}{n_{ij}!},n_{ij}=0,1,2,\ldots$$

Here, $\lambda_{ij}$ was defined as the mean (expected) value of $n_{ij}$ . Accordingly, $\lambda_{ij}=n_{i}p_{ij}$ . The relationship between mean value $\lambda_{ij}$ and covariate $x_{i}$ is generally modeled using a natural log link function, as follows:

$$\ln\left(\lambda_{ij}\right)=\beta_{0}+\beta_{i}x_{i}$$ (3)

where $\beta_{i}$ is the unknown regression parameter for drug i . We aimed to evaluate all ADRs ( $\left.j=1,\ldots ,J\right)$ when drug i is used. Considering that $x_{i}$ denotes the binary indicator for drug i , $x_{i}=1$ if the use of drug i is reported, and $x_{i}=0$ if the use of other drugs (excluding the i th drug) is reported. In this model, $\beta_{i}$ can be interpreted as the marginal effect of all ADRs using drug i compared to the use of other drugs. Thus, $\beta_{i}$ is a common effect that does not specify ADRs. Furthermore, $\exp\left(\beta_{i}\right)$ is the PRR for drug i that is not ADR specific. However, this interpretation of $\beta_{i}$ must be oversimplified and cannot detect the signal of a specific ADR.

Next, to assess a specific ADR, we included a random effect in Eq. 3, with the linear predictor $\eta_{ij}$ expressed as follows:

$$\eta_{ij}=\ln\left(\lambda_{ij}\right)=\beta_{0}+b_{0}+\left(\beta_{i}+b_{j}\right)x_{i}$$ (4)

where $b_{0}$ and $b_{j}$ are random effects for the intercept and j th ADR, respectively, assumed to follow normal distributions, $b_{0}∼N\left(0,\gamma_{0}\right)$ and $\left.b_{j}∼N\left(0,\right.\gamma_{j}\right)$ . Here, $\gamma_{0}$ and $\gamma_{j}$ are the variances of the random effects. Eq. 4, known as the Poisson mixed-effect model, was used to estimate the mean value $\lambda_{ij}$ for each drug and each ADR.

Based on Eq. 4, the linear predictor for each $x_{i}$ was

$$\ln\left(\lambda_{ij}\right)=\left\{\begin{array}{cc} \beta_{0}+b_{0}+\beta_{i}+b_{j}, & x_{i}=1\\ \beta_{0}+b_{0}, & x_{i}=0 \end{array}\right.$$

Thus, the PRR of ADR j for drug i is

$${\text{PRR}}_{ij}=\exp\left(\beta_{i}+b_{j}\right).$$

Using Eq. 4, we simultaneously estimated the PRRs of all ADRs (i.e., any j ) for drug i .

Extending Eq. 4 allowed the simultaneous evaluation effects mediated by multiple drugs; for example, consider a DDI in which two drugs ( i and $i^{\prime }$ ) are administered simultaneously. In this case, the linear predictor in Eq. 4 is expressed as follows:

$$\ln\left(\lambda_{ii^{\prime }j}\right)=\beta_{0}+b_{0}+\beta_{i}x_{i}+\beta_{i^{\prime }}x_{i^{\prime }}+\beta_{ii^{\prime }}x_{i}x_{i^{\prime }}+b_{ij}x_{i}+b_{i^{\prime }j}x_{i^{\prime }}+b_{ii^{\prime }j}x_{i}x_{i^{\prime }}$$

where $x_{i}=1$ if drug i was used, $x_{i}=0$ otherwise, $x_{i^{\prime }}=1$ if drug $i^{\prime }$ was used, and $x_{i^{\prime }}=0$ otherwise. $\beta_{i}$ , $\beta_{i^{\prime }}$ , and $\beta_{ii^{\prime }}$ are unknown regression parameters for $x_{i}$ , $x_{i^{\prime }}$ , and $x_{i}x_{i^{\prime }}$ , respectively; $b_{0}$ , $b_{ij}$ , $b_{i^{\prime }j}$ , and $b_{ii^{\prime }j}$ are random effects for intercepts, $x_{i}$ , $x_{i^{\prime }}$ , and $x_{i}x_{i^{\prime }}$ , respectively. Assuming that $b_{0}∼N\left(0,\gamma_{0}\right)$ , $b_{ij}∼N\left(0,\gamma_{i}\right)$ , $b_{i^{\prime }j}∼N\left(0,\gamma_{i^{\prime }}\right)$ , and $b_{ii^{\prime }j}∼N\left(0,\gamma_{ii^{\prime }}\right)$ , each linear predictor can be calculated as follows:

$$\ln\left(\lambda_{ii^{\prime }j}\right)=\left\{\begin{array}{cc} \beta_{0}+b_{0}, & x_{i}=0\text{ and }x_{i^{\prime }}=0\\ \beta_{0}+b_{0}+\beta_{i}+b_{ij\;}, & x_{i}=1\text{ and }x_{i^{\prime }}=0\\ \beta_{0}+b_{0}+\beta_{i^{\prime }}+b_{i^{\prime }j\;}, & x_{i}=0\text{ and }x_{i^{\prime }}=1\\ \beta_{0}+b_{0}+\beta_{i}+\beta_{i^{\prime }}+\beta_{ii^{\prime }}+b_{ij\;}+b_{i^{\prime }j\;}+b_{ii^{\prime }j}, & x_{i}=1\text{ and }x_{i^{\prime }}=1 \end{array}\right.$$

Thus, the PRRs of ADR j for drugs i and $i^{\prime }$ and the combined use of drugs i and $i^{\prime }$ are as follows:

$${\text{PRR}}_{ij}=\exp\left(\beta_{i}+b_{ij}\right),{\text{PRR}}_{i^{\prime }j}=\exp\left(\beta_{i^{\prime }}+b_{i^{\prime }j}\right),\text{and }{\text{PRR}}_{ii^{\prime }j}=\exp\left(\beta_{ii^{\prime }}+b_{ii^{\prime }j}\right),$$

Here, ${\text{PRR}}_{ij}$ is the PRR of ADR j for drug i , ${\text{PRR}}_{i^{\prime }j}$ is the PRR of ADR j for drug $i^{\prime }$ , and ${\text{PRR}}_{ii^{\prime }j}$ is the PRR of ADR j under the concomitant use of drugs i and $i^{\prime }$ . The proposed method could allow the detection of DDIs during the concurrent use of two or more drugs, as it allows for flexible modeling by including a statistical interaction term. The proposed method is based on a multiplicative model for DDI, whereas the criteria for detecting DDIs established by Norén et al. (2008) and Gosho et al. (2017) are based on an additive model for DDI (Thakrar et al., 2007).

The fixed and random effects in Eqs 3, 4 were estimated using the restricted pseudo-likelihood method (Wolfinger and O’Connell, 1993). The PRR and its 95% CI were estimated using the estimation of $\beta_{i}$ and $b_{ij}$ , ${\hat{\beta }}_{i}$ and ${\hat{b}}_{ij}$ and their variance estimates via pseudo-likelihood theory. Stroup (2013) provides a more detailed explanation regarding the theory of generalized mixed-effect models, such as Poisson and logistic mixed-effect models. If the lower limit of the 95% CI for the PRR was >1, the relationship between the target drug and the specific event was detected as a signal.

The simple PRR in Eq. 1 cannot be applied for signal detection when $n_{ij}=0$ , given that the 95% CI for PRR in Eq. 1 cannot be estimated when $n_{ij}=0$ . However, the proposed method could provide a 95% CI for the PRR estimated using Eq. 4 even when $n_{ij}=0$ .

### 2.4 Logistic mixed-effect model and ROR

The modeling strategies described in Section 2.3 can be easily applied to logistic regression analysis. We assumed that the random variable $n_{ij}$ follows the binomial distribution $n_{ij}\;∼\text{ Bin}\left(n_{i},p_{ij}\right)$ . As described in Section 2.3., the logistic mixed-effects model can be expressed as follows:

$$\eta_{ij}=\ln\frac{p_{ij}}{1-p_{ij}}=\beta_{0}+b_{0}+\left(\beta_{i}+b_{j}\right)x_{i}$$ (5)

Using Eq. 5, the linear predictor for each $x_{i}$ can be calculated as follows:

$$\ln\frac{p_{ij}}{1-p_{ij}}=\left\{\begin{array}{cc} \beta_{0}+b_{0}+\beta_{i}+b_{j}, & x_{i}=1\\ \beta_{0}+b_{0}, & x_{i}=0 \end{array}\right.$$

Thus, the ROR of ADR j for drug i is

$${\text{ROR}}_{ij}=\exp\left(\beta_{i}+b_{j}\right).$$

Based on Eq. 5, we could simultaneously estimate the RORs of all ADRs (i.e., any j ) for drug i .

Using the method described in Section 2.3, Eq. 5 was used to simultaneously evaluate the effects of multiple drugs. For example, consider a DDI in which two drugs are administered simultaneously. The linear predictor in Eq. 5 is expressed as follows:

$$\ln\frac{p_{ii^{\prime }j}}{1-p_{ii^{\prime }j}}=\beta_{0}+b_{0}+\beta_{i}x_{i}+\beta_{i^{\prime }}x_{i^{\prime }}+\beta_{ii^{\prime }}x_{i}x_{i^{\prime }}+b_{ij}x_{i}+b_{i^{\prime }j}x_{i^{\prime }}+b_{ii^{\prime }j}x_{i}x_{i^{\prime }}$$

Thus, the RORs of ADR j for drugs i and $i^{\prime }$ and the combined use of drugs i and $i^{\prime }$ are as follows:

$${\text{ROR}}_{ij}=\exp\left(\beta_{i}+b_{ij}\right),{\text{ROR}}_{i^{\prime }j}=\exp\left(\beta_{i^{\prime }}+b_{i^{\prime }j}\right),\text{and }{\text{ROR}}_{ii^{\prime }j}=\exp\left(\beta_{ii^{\prime }}+b_{ii^{\prime }j}\right).$$

${\text{ROR}}_{ij}$ is the ROR of ADR j for drug i , ${\text{ROR}}_{i^{\prime }j}$ is the ROR of ADR j for drug $i^{\prime }$ , and ${\text{ROR}}_{ii^{\prime }j}$ is the ROR of ADR j during the concomitant use of drugs i and $i^{\prime }$ . If the lower limit of the 95% CI for the PRR was >1, the relationship between the target drug and the specific event was detected as a signal.

If $p_{ij}$ is small, the ROR well-approximated the PRR. Given that $p_{ij}$ is usually small in signal detection analyses, ROR and PRR did not differ significantly in almost all cases.

## 3 Application

We analyzed the FDA Adverse Event Reporting System (FAERS), a well-known database comprising adverse event reports designed to support the FDA’s post-marketing drug safety surveillance program. FAERS includes seven data files: demographics (e.g., sex and age), drugs (e.g., drug name and route of drug administration), reaction (e.g., terms of an adverse event), outcome (patient outcome), report source (code for the source of the report), therapy date (e.g., the date on which the therapy was started and stopped), and indications for use. Adverse events are determined using the Medical Dictionary for Regulatory Activities (MedDRA) as the preferred term.

Recently, sodium glucose-linked transporter 2 (SGLT2) inhibitors, a class of oral antidiabetic drugs, have been widely used to treat type 2 diabetes. The FDA approved canagliflozin as the first SGLT2 inhibitor for treating type 2 diabetes in 2013 (Mosley et al., 2015). Since then, six SGLT2 inhibitors have been approved in the US and Japan. The proposed logistic mixed-effect and Poisson mixed-effect models were applied to the signal detection analysis of these SGLT2 inhibitors for potential ADRs in two scenarios: 1) signal detection for one drug and 2) DDIs following the concomitant use of two drugs, as well as a simulation study.

Data files were downloaded from the FDA website (https://fis.fda.gov/extensions/FPD-QDE-FAERS/FPD-QDE-FAERS.html) and analyzed between 2014 Q1 and 2022 Q4 after the launch of SGLT2 inhibitors. The analyses included records describing 13,344,838 patient characteristics, 54,869,999 drug properties, and 43,029,283 reactions/events. All analyses were performed using SAS software version 9.4 (SAS Institute, Cary, NC). The SAS code is provided in the Supplementary Material.

### 3.1 Scenario 1 (single drug)

We applied the two proposed models to the FAERS database to screen for ADRs when seven SGLT2 inhibitors (canagliflozin, empagliflozin, ipragliflozin, dapagliflozin, tofogliflozin, luseogliflozin, and ertugliflozin) were used. As a reference, we also applied the traditional ROR and PRR to the database.

A list of ADRs determined as signals using the proposed methods is presented in Supplementary Table S1. The total run time of the analysis was 30 min (2.2 GHz Intel Xeon processor with 64 GB memory). When the lower limit of the 95% CI for the proposed ROR and PRR was greater than 1, the ADR was considered detected. The results of the proposed ROR were similar to those of the proposed PRR owing to the low reporting rate. In addition, the ADRs detected using the proposed methods were similar to those detected using traditional methods. As numerous ADRs were detected (Supplementary Table S1), we summarized the ADRs detected with three or more SGLT2 inhibitors (Table 2). We only presented ROR results because there was no significant difference between PRR and ROR. In addition, owing to space limitation, only ADRs classified as “metabolism and nutrition disorders metabolism” in the system organ class (SOC) of MedDRA or recognized in the package insert of SGLT2 inhibitors are listed in Table 2.

**TABLE 2:** ADRs detected with three or more kinds of SGLT2 inhibitors, and the lower limit of 95% CI for ROR calculated using the proposed model in Scenario 1 (single drug).

Detected ADR	SGLT2 inhibitors						
	Canagliflozin ( $n_{i∙}=3933$ )	Empagliflozin ( $n_{i∙}=36966$ )	Ipragliflozin ( $n_{i∙}=553$ )	Dapagliflozin ( $n_{i∙}=14054$ )	Tofogliflozin ( $n_{i∙}=147$ )	Luseogliflozin ( $n_{i∙}=133$ )	Ertugliflozin ( $n_{i∙}=759$ )
Metabolism and nutrition disorders in system organ class							
Hypoglycemia	**7.35**	**4.18**	**8.55**	**10.23**	**1.61**	0.52	**2.50**
Diabetic ketoacidosis	**75.67**	-	**10.77**	**118.63**	**18.05**	**5.65**	**30.58**
Euglycemic diabetic ketoacidosis	**140.42**	**393.33**	**10.06**	**236.20**	**82.39**	**21.65**	**52.34**
Ketoacidosis	**76.20**	**131.99**	**32.16**	**171.53**	**2.88**	**3.19**	**17.90**
Diabetic ketosis	**14.97**	**39.91**	**16.05**	**189.73**	-	**226.11**	-
Ketosis	**127.62**	**74.74**	**22.47**	**217.34**	**24.72**	-	**49.15**
Dehydration	**3.17**	**3.71**	**4.04**	**3.95**	**5.23**	0.18	0.98
Polydipsia	**7.78**	**10.90**	-	**6.22**	-	-	-
Diabetes mellitus	**6.35**	**2.34**	**7.56**	**3.05**	**4.00**	**1.05**	0.06
Type 1 diabetes mellitus	0.15	**1.40**	1.00	**3.47**	**3.75**	-	-
Type 2 diabetes mellitus	**14.01**	**1.18**	**1.42**	**1.82**	0.53	-	0.35
Diabetes mellitus inadequate control	**13.05**	**6.90**	**20.31**	**18.71**	**28.29**	**13.81**	**5.37**
Hyperglycemia	**17.07**	**5.64**	**4.40**	**13.85**	**14.06**	**9.57**	**1.20**
Insulin resistance	-	**4.37**	-	**1.91**	**9.90**	-	-
Diabetic metabolic decompensation	-	**10.88**	-	**53.40**	-	-	**55.74**
Acetonemia	**38.53**	**75.23**	-	**51.36**	-	-	-
Acidosis	**8.46**	**9.47**	**2.84**	**6.99**	-	-	-
Decreased appetite	0.79	**1.42**	**1.46**	**1.33**	**1.15**	**2.95**	0.29
Dyslipidemia	**5.58**	**1.08**	-	**5.65**	-	-	-
Fluid intake reduced	**1.11**	**1.66**	-	**3.87**	**4.46**	-	-
Hyperglycemic hyperosmolar nonketotic syndrome	**5.43**	**13.97**	**19.85**	**38.27**	-	-	-
Hyperkalemia	**1.97**	**2.65**	**1.73**	**5.86**	0.64	-	0.43
Hyperlipidemia	0.55	**1.93**	-	**2.05**	**2.23**	**8.36**	-
Hypernatremia	**5.17**	**3.27**	-	**11.06**	-	-	0.88
Hypertriglyceridemia	**3.87**	**1.41**	**3.45**	**1.27**	-	-	-
Hyperuricemia	**1.67**	**1.16**	**21.30**	**7.64**	-	-	-
Hypokalemia	**1.61**	**1.09**	0.80	**1.79**	-	-	0.31
Hypophagia	**2.30**	**2.36**	0.21	**1.24**	-	-	-
Hypovolemia	**7.67**	**11.50**	-	**6.91**	-	-	0.90
Obesity	**1.49**	0.88	**3.38**	**3.39**	**1.25**	-	0.84
Starvation	**1.23**	**2.88**	-	**6.14**	-	-	-
ADRs recognized in the package insert of SGLT2 inhibitors							
Coronary artery stenosis	**7.02**	**3.23**	**5.33**	**14.71**	**5.87**	-	-
Cerebral infarction	**19.66**	**2.74**	**24.66**	**8.60**	**8.84**	**6.27**	-
Thrombotic cerebral infarction	**1.75**	**5.82**	**78.94**	**38.16**	-	**49.76**	-
Embolic cerebral infarction	**1.07**	**1.57**	**7.39**	**1.01**	-	-	-
Cerebellar infarction	**5.43**	**1.54**	**3.79**	**4.06**	-	-	-
Lacunar infarction	**7.76**	**3.50**	**3.02**	**7.96**	-	**42.40**	**2.30**
Brain stem infarction	**5.68**	**1.68**	**6.12**	**13.76**	-	-	-
Carotid artery stenosis	**9.79**	**2.36**	-	**2.34**	-	**9.05**	-
Ketonuria	**72.99**	**86.02**	-	**211.69**	-	-	**17.74**
Nocturia	**3.06**	**2.83**	0.43	**1.38**	-	-	0.32
Polyuria	**13.18**	**8.70**	0.65	**19.31**	-	-	**3.13**
Balanoposthitis	**28.25**	**45.10**	-	**70.31**	-	-	**4.77**
Pruritus genital	**14.46**	**25.45**	**9.53**	**36.46**	**10.50**	-	**2.14**
Vulvovaginal pruritus	0.98	**14.40**	-	**7.15**	-	-	**2.65**
Genital discomfort	-	**19.05**	-	**17.82**	-	-	**46.07**
Penile erythema	**2.05**	**20.82**	-	**3.70**	-	-	**10.71**
Penile pain	**5.33**	**2.73**	-	**2.60**	-	-	-
Scrotal swelling	**2.99**	**5.74**	-	**1.61**	-	-	-
Vulvovaginal erythema	-	**4.63**	-	**3.34**	-	-	**15.02**
Vulvovaginal swelling	0.39	**2.90**	-	**1.50**	-	-	**6.75**
Pyelonephritis	**6.73**	**3.50**	**5.88**	**8.55**	**7.39**	**8.16**	**1.46**
Pyelonephritis acute	**20.63**	**3.21**	-	**14.17**	**9.69**	-	-
Emphysematous pyelonephritis	**12.83**	**99.69**	**89.65**	**1.09**	-	**371.02**	-
Sepsis	**1.29**	**2.22**	0.68	**2.09**	**1.19**	0.20	0.80
Septic shock	**1.44**	**1.51**	-	**2.21**	0.50	-	0.33
Fournier’s gangrene	**55.03**	**249.97**	-	**59.85**	-	-	**71.49**
Gangrene	**4.53**	**7.10**	-	**5.56**	-	-	**6.54**
Necrotizing fasciitis	**5.33**	**24.27**	-	**22.61**	-	-	**4.20**
Necrotizing soft tissue infection	**9.74**	**28.54**	-	**2.75**	-	-	**50.33**
Diabetic gangrene	**96.42**	**3.43**	-	**6.53**	-	-	-
Thirst	**4.00**	**8.64**	**1.99**	**6.26**	-	-	**6.04**
Amputation	**28.07**	**5.69**	-	**2.39**	-	-	-
Foot amputation	**6.43**	**7.02**	-	**5.93**	-	-	**8.13**
Leg amputation	**6.05**	**5.65**	-	**3.55**	-	-	-
Toe amputation	**10.72**	**9.57**	-	**8.26**	-	-	**2.18**

ADR, adverse drug reaction; CI, confidence interval; ROR, reporting odds ratio; SGLT2, sodium glucose-linked transporter 2.

- not reported; bold, detected ADR; $n_{i∙}$ , the number of the target drug i reported.

In the current analysis, hypoglycemia, ketoacidosis, and several infarctions, all well-known ADRs of SGLT2 inhibitors, were detected with almost all SGLT2 inhibitors. Euglycemic diabetic ketoacidosis, ketoacidosis, and pyelonephritis were detected with all seven SGLT2 inhibitors. The detection results of the proposed methods were similar to those observed with the traditional ROR and PRR (Supplementary Table S1).

### 3.2 Scenario 2 (DDI)

Patients with diabetes frequently coadminister SGLT2 inhibitors with glimepiride, a sulfonylurea that stimulates pancreatic *β* cells to release insulin. Accordingly, the proposed models were applied to assess DDIs between seven SGLT2 inhibitors and glimepiride.

A list of ADRs determined as signals using the proposed methods is presented in Supplementary Table S2. The number of drugs reported is also shown in Supplementary Table S3. The total run time of the analysis was 232 min (2.2 GHz Intel Xeon processor with 64 GB memory). For the proposed PRR and ROR, an ADR was considered to be detected when the lower limits of 95% CI were >1. The results of the proposed ROR were similar to those of the proposed PRR. As observed in Scenario 1, Table 3 presents a list of ADRs detected with two or more types of SGLT2 inhibitors using the proposed methods. Only ROR results are presented, given the absence of any significant difference between the PRR and ROR. Owing to space limitations, only ADRs classified as “metabolism and nutrition disorders metabolism,” “cardiac disorders,” “nervous system disorders,” “renal and urinary disorders,” “reproductive system and breast disorders,” “infections and infestations,” and “surgical and medical procedures” in SOC of MedDRA are listed in Table 3. These SOC classes include ADRs that are likely to occur with the use of SGLT2 inhibitors.

**TABLE 3:** ADRs detected with two or more kinds of SGLT2 inhibitors with glimepiride and the lower limit of 95% CI for ROR calculated using the proposed model in Scenario 2 (DDIs).

Detected ADR	Concomitant use of glimepiride and						
	Canagliflozin ( $n_{ii^{\prime }∙}=467$ )	Empagliflozin ( $n_{ii^{\prime }∙}=1346$ )	Ipragliflozin ( $n_{ii^{\prime }∙}=116$ )	Dapagliflozin ( $n_{ii^{\prime }∙}=780$ )	Tofogliflozin ( $n_{ii^{\prime }∙}=26$ )	Luseogliflozin ( $n_{ii^{\prime }∙}=20$ )	Ertugliflozin ( $n_{ii^{\prime }∙}=34$ )
Metabolism and nutrition disorders							
Hypoglycemia	**9.78**	**3.67**	**8.40**	-	-	**1.45**	-
Diabetic ketoacidosis	**31.63**	-	**5.62**	-	-	-	**1.40**
Euglycemic diabetic ketoacidosis	**70.15**	-	**3.69**	**86.16**	-	-	-
Ketoacidosis	**24.65**	**15.48**	-	**39.40**	-	-	**30.90**
Diabetic ketosis	-	**22.14**	-	**84.30**	-	-	-
Ketosis	**145.37**	**105.86**	-	**17.17**	**56.46**	-	-
Abnormal loss of weight	-	**10.64**	-	**10.87**	-	-	-
Dehydration	**5.07**	-	0.52	**4.58**	-	0.46	0.28
Diabetes mellitus	0.52	**2.89**	0.93	**2.44**	0.63	-	-
Type 2 diabetes mellitus	0.46	**2.06**	-	**2.62**	-	-	**-**
Diabetes mellitus inadequate control	**6.98**	**12.37**	0.79	**28.45**	**-**	**3.66**	**2.24**
Diabetic metabolic decompensation	**-**	0.85	**-**	**261.80**	**-**	**-**	**459.27**
Hyperglycemia	**1.82**	**4.89**	**-**	**-**	**1.37**	**1.78**	**-**
Hypercholesterolemia	**-**	**6.58**	**-**	**1.46**	**-**	**-**	**-**
Hyperuricemia	**-**	**6.49**	**14.61**	**-**	**-**	**-**	**-**
Increased appetite	**1.06**	0.09	**-**	**2.16**	**-**	**-**	**-**
Lactic acidosis	0.64	**1.76**	**2.40**	**21.13**	**1.61**	**2.10**	**-**
Metabolic acidosis	**4.91**	**-**	**-**	**1.28**	**-**	**-**	**-**
Obesity	**-**	**1.30**	**-**	**1.38**	**-**	**-**	**-**
Cardiac disorders							
Coronary artery disease	0.16	**1.25**	**-**	**2.13**	**-**	**-**	**1.50**
Coronary artery occlusion	**-**	**1.21**	**-**	**3.29**	**-**	**-**	**-**
Coronary artery stenosis	**49.45**	**3.89**	**3.70**	**3.21**	**-**	**-**	**-**
Acute myocardial infarction	**5.26**	**7.37**	**-**	**8.11**	**-**	**-**	**1.35**
Angina pectoris	**5.22**	**7.76**	0.47	**3.12**	**-**	**-**	**-**
Angina unstable	0.67	**1.15**	**-**	**1.93**	**-**	**-**	**-**
Atrial fibrillation	**4.42**	0.82	0.69	**1.41**	**-**	**-**	**-**
Cardiac failure	**3.33**	**1.30**	0.16	**1.52**	**8.11**	**-**	**-**
Myocardial infarction	**1.64**	**1.85**	0.42	**1.22**	**-**	**-**	**-**
Myocardial ischemia	**3.83**	**3.08**	**1.31**	0.24	**-**	**-**	**-**
Ventricular extrasystoles	**4.73**	**3.81**	**-**	**-**	**-**	**-**	**-**
Ventricular fibrillation	**-**	**13.08**	**-**	**4.18**	**-**	**-**	**-**
Tricuspid valve incompetence	**-**	**2.30**	**-**	**3.92**	**-**	**-**	**-**
Nervous system disorders							
Cerebral infarction	**34.52**	**10.56**	**2.88**	**-**	**-**	**15.73**	**-**
Cerebellar infarction	**12.36**	**9.24**	**8.84**	**-**	**-**	**-**	**-**
Lacunar infarction	**-**	0.84	**-**	**12.79**	**-**	**207.12**	**-**
Brain stem infarction	**-**	**7.33**	**14.41**	**41.61**	**-**	**-**	**-**
Diabetic neuropathy	**-**	**1.52**	**-**	**8.46**	**-**	**-**	**-**
Altered state of consciousness	0.19	**2.45**	**17.03**	**5.06**	**-**	**-**	**-**
Cervicobrachial syndrome	**79.17**	**13.67**	**-**	**-**	**-**	**-**	**-**
Renal and urinary disorders							
Ketonuria	**-**	**35.49**	**-**	**37.32**	**-**	**-**	**-**
Nocturia	**1.38**	**1.04**	**-**	0.84	**-**	**-**	**-**
Pollakiuria	**2.58**	**-**	**-**	**1.94**	**-**	**-**	**-**
Polyuria	**4.49**	**6.01**	**-**	**16.92**	**-**	**-**	**-**
Renal impairment	**6.84**	**2.19**	**2.66**	**1.75**	**-**	**-**	**-**
Acute kidney injury	**1.35**	**-**	**2.07**	**2.27**	0.21	**-**	0.99
Nephropathy	**1.32**	**1.58**	**-**	**-**	**-**	**-**	**-**
Renal failure	**1.07**	**1.01**	0.08	0.23	**-**	**-**	0.23
Dysuria	0.47	**2.12**	**-**	**2.67**	**-**	**-**	**-**
Hematuria	**-**	**1.30**	0.34	**1.78**	**-**	**-**	**-**
Urinary incontinence	**1.27**	0.73	**-**	**1.75**	**-**	**-**	**-**
Urinary retention	**5.24**	0.20	**-**	**6.05**	**-**	**-**	**-**
Reproductive system and breast disorders							
Balanoposthitis	**4.44**	**17.71**	**-**	**13.76**	**-**	**-**	**-**
Pruritus genital	**-**	**16.72**	**-**	**19.61**	**23.53**	**-**	**-**
Vulvovaginal pruritus	0.73	**7.91**	**-**	**2.12**	**-**	**-**	**-**
Testicular pain	**1.20**	**2.07**	**-**	**38.05**	**-**	**-**	**-**
Benign prostatic hyperplasia	**6.47**	**1.12**	**-**	0.41	**-**	**-**	**-**
Infections and infestations							
Pyelonephritis	**1.89**	0.69	**7.17**	**5.50**	**4.82**	**-**	**-**
Pyelonephritis acute	**8.58**	**6.41**	**-**	**49.32**	**21.72**	**-**	**-**
Sepsis	0.76	**1.64**	**-**	**1.57**	**-**	**-**	0.32
Septic shock	**3.42**	0.52	**-**	**2.48**	**1.10**	**-**	**-**
*Escherichia* sepsis	**-**	**-**	**85.55**	**3.88**	**-**	**-**	**-**
Fournier’s gangrene	**1.52**	**93.96**	**-**	**21.73**	**-**	**-**	**-**
Gangrene	**-**	**-**	**-**	**8.33**	**-**	**-**	**35.25**
Necrotizing fasciitis	**1.17**	**2.14**	**-**	**11.50**	**-**	**-**	**-**
Scrotal abscess	**-**	**16.64**	**-**	**26.23**	**-**	**-**	**-**
Fungal infection	**-**	**8.85**	**-**	0.30	**-**	**-**	**13.78**
Gastroenteritis	**5.24**	0.09	0.78	**4.14**	**-**	**-**	**-**
Pneumonia aspiration	**2.44**	**-**	**-**	**6.38**	**-**	**-**	**-**
Surgical and medical procedures							
Leg amputation	**4.43**	**1.63**	**-**	**2.70**	**-**	**-**	**-**

ADR, adverse drug reaction; CI, confidence interval; DDI, drug-drug interaction; ROR, reporting odds ratio; SGLT2, sodium glucose-linked transporter 2.

- not reported; bold, detected ADR; $n_{ii^{\prime }∙}$ , the number of the combined uses of drug i and drug $i^{\prime }$ reported.

In addition, ketosis-related ADRs were frequently detected following the concomitant use of glimepiride and several SGLT2 inhibitors. Coronary artery stenosis, acute myocardial infarction, cardiac failure, cerebral infarction, renal impairment, and acute pyelonephritis were detected in patients treated with four SGLT2 inhibitors (Table 3).

## 4 Simulation study

We examined the performance of the proposed method using a simulation study. We calculated the ROR and PRR using the logistic mixed-effect and Poisson mixed-effect models, respectively, as defined in Section 2.

The performance was evaluated in terms of sensitivity and false-positive rates. Sensitivity is the proportion of correctly identified signals, whereas the false-positive rate is the proportion of falsely detected signals. In this section, we considered two simulations: 1) signal detection for one drug and 2) DDI for the concomitant use of two drugs.

### 4.1 Data generation

Data generation was repeated 1,000 times for each setting. The number of ADR types was set as J=100,500 .

#### 4.1.1 Simulation 1 (single drug)

Signal detection was considered when only one drug was administered. The number of ADRs reported for each drug was set to n=10,000,000 . For drug 1 ( i=1 ), the number of prescriptions with drug 1 was set to $n_{1\cdot }=10,000,50,000$ . In Scenario 1, we investigated the false-positive rate of the proposed method, setting the incidence probabilities of ADR j to $p_{1j}=p_{1^{-}j}=0.05,0.1,0.2$ (%). Here, $p_{1^{-}j}$ is the incidence probability of ADR j when drug 1 is not used (see Table 1). In Scenario 2, we examined the sensitivity by setting $\left(p_{1^{-}j},p_{1j})=\left(0.05,0.075\right),\left(0.05,0.1\right),\left(0.05,0.125\right),\left(0.1,0.15\right),\left(0.1,0.2\right),(0.1,0.25\right)$ (%). In both scenarios, the values of $n_{1j}$ and $n_{j}-n_{1j}$ were independently generated by the binomial distributions Bin( $\left.n_{1\cdot },p_{1j}\right)$ and Bin( $\left.n-n_{1\cdot },p_{1^{-}j}\right)$ , respectively.

#### 4.1.2 Simulation 2 (DDI)

**FIGURE 1:**
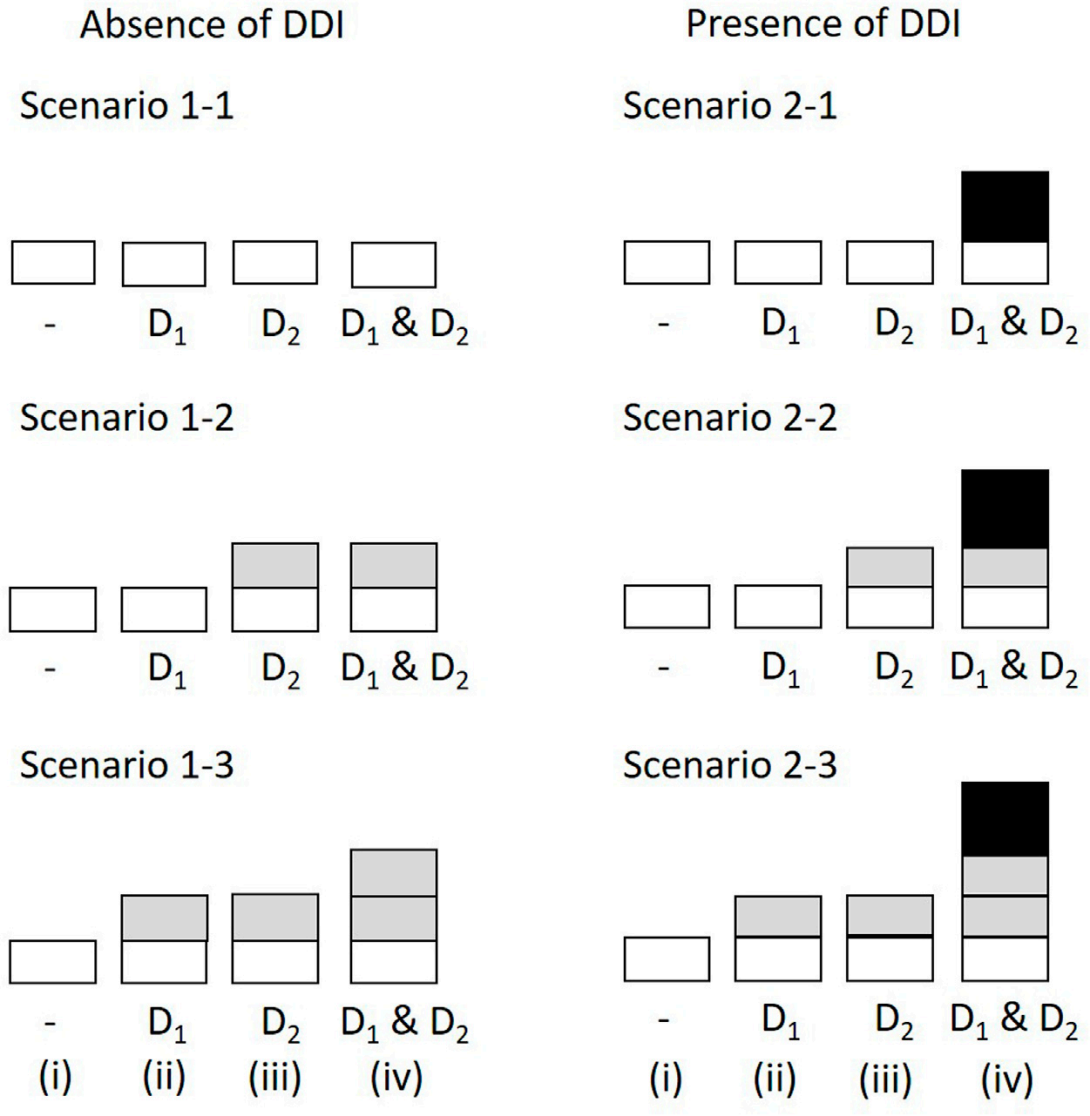
Simulation scenarios and settings. The height of *bars* correspond to the incidence probability of ADR A (i) in the absence of both D_1_ and D_2_; (ii) with D_1_ but not D_2_; (iii) with D_2_ but not D_1_; (iv) with D_1_ and D_2_. The *shades* correspond to the marginal relative probability of ADR A (*lightest*), the increased frequency attributable to D_1_, the increased frequency attributable to D_2_, and the increased incidence probability attributable to an interaction between D_1_ and D_2_ (*darkest*). The darkest bars indicate the DDI effect. ADR, adverse drug reaction; DDI, drug-drug interaction.

We evaluated DDIs with the concomitant use of drugs 1 (D_1_) and 2 (D_2_) by assuming the number of prescriptions in the absence of both drugs, the presence of either D_1_ or D_2_, and the presence of both drugs to be 10,000,000, 100,000, and 10,000, respectively (Table 4). The incidence probabilities in Table 4 vary depending on the simulation scenario. In Scenario 1, we determined the false-positive rate of the proposed method. In this case, no DDIs were observed. We then set (1–1) $p_{00}=p_{10}=p_{01}=p_{11}$ ; (1–2) $p_{00}=p_{10}<\;p_{01}=p_{11}$ ; and (1–3) $p_{00}<p_{10}=p_{01}<\;p_{11}$ (Figure 1). An additional effect was observed between drugs 1 and 2 in (1–2) and (1–3), although no interaction was observed under the multiplicative assumption because $p_{00}p_{11}/\left(p_{10}p_{01}\right)=1$ . In Scenario 2, we investigated the sensitivity and detected a positive DDI because $p_{00}p_{11}/\left(p_{10}p_{01}\right)>1$ . Under this assumption, we set (2–1) $p_{00}=p_{10}=p_{01}<p_{11}$ , (2–2) $p_{00}=p_{10}<\;p_{01}<p_{11}$ , and (2–3) $p_{00}<p_{10}=p_{01}<p_{11}$ (Figure 1). The details of these settings are shown in Figure 1 and described in the Results section. In both scenarios, the values of $n_{00}$ , $n_{10}$ , $n_{01}$ , and $n_{11}$ were independently generated using binomial distributions. As a competitor (henceforth, the existing method), we calculated the PRR and ROR using simple Poisson and logistic models, including two factors D_1_ and D_2_, and the interaction term for each ADR, respectively (van Puijenbroek et al., 1999).

**TABLE 4:** Four-by-two contingency table summarizing the specific ADR reported with the target drugs for evaluating DDIs.

Number of events (incidence probability for j th ADR)	Specific ADR j	All others	Total
Neither drug 1 nor drug 2	$n_{00}$ ( $\left.p_{00}\right)$	$10,000,000-n_{00}$	10,000,000
Only drug 1	$n_{10}$ ( $\left.p_{10}\right)$	$100,000-n_{10}$	100,000
Only drug 2	$n_{01}$ ( $\left.p_{01}\right)$	$100,000-n_{01}$	100,000
drug 1 and drug 2	$n_{11}$ ( $\left.p_{11}\right)$	$10,000-n_{11}$	10,000

ADR, adverse drug reaction; DDIs, drug-drug interactions.

### 4.2 Results

#### 4.2.1 Simulation 1 (single drug)

Table 5 presents the false-positive rate in Scenario 1 and the sensitivity in Scenario 2 when the traditional and proposed PRR and ROR are applied under J=100 . The simulation results under J=500 are presented in Supplementary Table S4. At the top of Table 5, the false-positive rates of the proposed PRR and ROR were similar to those of the traditional PRR and ROR across all simulation settings. Additionally, false-positive rates of the PRR and ROR differed minimally. The false-positive rate for the proposed method was not dependent on the incidence probability or the number of ADR types. As shown in Table 5 (bottom), the sensitivities of the proposed PRR and ROR were similar to those of the traditional PRR and ROR for all simulation settings. Furthermore, the false-positive rate of PRR and ROR differed minimally.

**TABLE 5:** Simulation results in Scenarios 1 and 2 with single-dose settings (the number of ADR types = 100).

*p*_1-*j*_ (%)	*p*_1*j*_ (%)	Proposed method		Traditional method	
		PRR	ROR	PRR	ROR
False-positive rate					
0.05	0.05	3.27	3.27	3.27	3.27
0.1	0.1	2.04	2.04	2.05	2.05
0.2	0.2	2.78	2.79	2.80	2.80
0.05	0.05	2.56	2.56	2.59	2.59
0.1	0.1	2.56	2.56	2.57	2.57
0.2	0.2	2.58	2.59	2.60	2.60
Sensitivity					
0.05	0.075	23.6	23.6	23.6	23.6
	0.1	56.5	56.5	56.5	56.5
	0.125	80.2	80.2	80.2	80.2
0.1	0.15	35.1	35.1	35.2	35.1
	0.2	78.4	78.4	78.4	78.4
	0.25	95.6	95.6	95.6	95.6

ADR, adverse drug reaction; DDIs, drug-drug interactions; PRR, proportional reporting rate; ROR, reporting odds ratio.

#### 4.2.2 Simulation 2 (DDI)

Table 6 presents the false-positive rate in Scenario 1 and the sensitivity in Scenario 2 when the proposed PRR and ROR were applied under J=100 . The simulation results under J=500 are presented in Supplementary Table S5. At the top of Table 6, the false-positive rate for DDIs using the proposed PRR and ROR was generally controlled at a nominal significance level of 5%. The false-positive rates of the proposed PRR and ROR were similar to those of the PRR and ROR derived using existing methods across all simulation settings. In addition, the false-positive rates of the PRR and ROR differed minimally. The false-positive rate for the proposed method was not dependent on the incidence probability or the number of ADR types. As shown in Table 6 (bottom), there was minimal difference in the sensitivity between PRR and ROR as a false-positive rate. The sensitivities of the proposed PRR and ROR were also similar to those of the PRR and ROR from the existing method across all simulation settings. Although the sensitivity of PRR and ROR increased as the incidence probability increased, the sensitivity was not dependent on the number of ADR types.

**TABLE 6:** Simulation results in Scenarios 1 and 2 with DDI settings (the number of ADR types = 100).

Scenario	*p*_00_ (%)	*p*_10_ (%)	*p*_01_ (%)	*p*_11_ (%)	Proposed method		Existing method	
					PRR	ROR	PRR	ROR
Scenario 1: false-positive rate (%)								
1–1	0.05	0.05	0.05	0.05	5.29	5.30	5.29	5.34
	0.1	0.1	0.1	0.1	3.86	3.87	3.96	4.03
	0.25	0.25	0.25	0.25	3.25	3.27	3.37	3.48
1–2	0.025	0.025	0.05	0.05	4.53	4.54	4.52	4.55
	0.05	0.05	0.1	0.1	3.48	3.48	3.59	3.64
	0.1	0.1	0.25	0.25	3.01	3.11	3.12	3.18
1–3	0.01	0.025	0.025	0.0625	3.35	3.35	3.30	3.33
	0.025	0.05	0.05	0.1	3.25	3.26	3.33	3.35
	0.05	0.1	0.1	0.2	3.04	3.06	3.13	3.17
Scenario 2: sensitivity (%)								
2–1	0.025	0.025	0.025	0.05	36.7	36.7	36.5	36.6
	0.05	0.05	0.05	0.1	53.9	53.9	54.1	54.3
	0.1	0.1	0.1	0.2	75.9	76.0	76.1	76.2
2–2	0.01	0.01	0.025	0.05	27.3	27.3	27.2	27.3
	0.025	0.025	0.05	0.1	48.0	48.1	47.7	47.9
	0.05	0.05	0.1	0.2	70.5	70.5	70.9	71.1
2–3	0.01	0.015	0.015	0.045	28.0	28.0	28.0	28.0
	0.02	0.03	0.03	0.09	45.1	45.2	44.9	45.0
	0.04	0.06	0.06	0.18	67.1	67.2	67.4	67.6

ADR, adverse drug reaction; DDIs, drug-drug interactions; PRR, proportional reporting rate; ROR, reporting odds ratio.

Simulation Scenario 1 (absence of DDI): 1–1, $p_{00}=p_{10}=p_{01}=p_{11}$ ; 1–2, $p_{00}=p_{10}<\;p_{01}=p_{11}$ ; 1–3 $p_{00}<p_{10}=p_{01}<\;p_{11}$ .

Simulation Scenario 2 (presence of DDI): 2–1, $p_{00}=p_{10}=p_{01}<p_{11}$ ; 2–2, $p_{00}=p_{10}<\;p_{01}<p_{11}$ ; 2–3, $p_{00}<p_{10}=p_{01}<p_{11}$

## 5 Discussion

Herein, we proposed a new signal detection method within the framework of a generalized mixed-effect model. The proposed models can directly estimate the PRR and ROR, which are used worldwide to detect signals in spontaneous reporting systems. In terms of the advantages, the proposed method can allow the simultaneous evaluation of several ADRs using multiple drugs. The proposed method is suitable for signal detection because ADRs should be comprehensively and efficiently screened in post-marketing drug safety surveillance. Our study also found that the PRR and ROR calculated using the proposed model were almost identical to the traditional PRR and ROR. While the traditional PRR and ROR can only be calculated in the presence of one drug, the proposed method can be applied to multiple drugs and is a more generalized and convenient method.

For screening ADRs in spontaneous reporting systems, the Medicines and Healthcare Products Regulatory Agency adopts the traditional PRR, and the Netherlands Pharmacovigilance Center and the Pharmaceutical and Medical Devices Agency in Japan employ the traditional ROR (Noguchi et al., 2021). The proposed method also provides the PRR and ORR, and it can be interpreted similarly to the results of traditional methods routinely used by the regulatory authorities. Although the criterion established by Norén et al. (Norén et al., 2008) would be the most widely used for detecting DDIs, the method proposed in the current study is more convenient for practical applications, given that ADRs from “single use of a drug” and “concomitant use of drugs” can be uniformly evaluated using one methodology. Thus, we anticipate that the proposed method will become one of the most useful applications in drug safety surveillance in the future.

However, some ingenuity is required to construct a generalized mixed-effects model. For example, the model may lead to convergence problems in numerical optimization when many drugs are included in the model as factors. Specifically, we cannot obtain PRR and ROR estimates when the constructed model is markedly complicated. In this case, the model is simplified. In addition, the calculation to obtain parameter estimates may be prolonged in the presence of numerous ADRs and drug types.

Several limitations need to be cautiously considered when undertaking signal detection analyses. For example, only observed ADRs are registered in spontaneous reporting systems databases, resulting in underreporting bias (Noguchi et al., 2021). Furthermore, the incidence rate for ADRs cannot be calculated because databases collect only patient information with the ADR (Tada and Gosho, 2022). Moreover, even if the patients are actually taking multiple drugs, some drug information might be missing. Therefore, the measures for detecting DDI tend to be underestimated (Norén et al., 2008). These limitations are inherent to databases and cannot be overcome even when using the proposed method. Although signal detection analysis fails to establish definite conclusions regarding the association between ADRs and target drugs due to the limitations, the analysis results generate hypotheses about the association.

## Data Availability

Publicly available datasets were analyzed in this study. This data can be found here: Publicly available datasets were analyzed in this study. This data can be found here: The following publicly available datasets were used in this study: https://fis.fda.gov/extensions/FPD-QDE-FAERS/FPD-QDE-FAERS.html.

## Abbreviations

ADR, adverse drug reaction; BCPNN, Bayesian confidence propagation neural network; CI, confidence interval; DDI, drug-drug interaction; FAERS, FDA adverse event reporting system; FDA, food and drug administration; MedDRA, medical dictionary for regulatory activity; MGPS, multi-item gamma poisson shrinker; PRR, proportional reporting rate; ROR, reporting odds ratio; SGLT2, sodium glucose-linked transporter 2; SOC, system organ class.
